# PGC‐1α inhibits polyamine metabolism in Cyclin E1‐driven ovarian cancer

**DOI:** 10.1002/cam4.2637

**Published:** 2019-10-27

**Authors:** Ting Guo, Bin Li, Chao Gu, Xiuying Chen, Mengxin Han, Xiaocheng Liu, Congjian Xu

**Affiliations:** ^1^ Department of Gynecology Obstetrics and Gynecology Hospital of Fudan University Shanghai PR China

**Keywords:** CCNE1, High‐grade serous ovarian cancer, polyamine metabolism

## Abstract

**Aim:**

Cyclin E1‐driven ovarian cancer (OvCa) is characterized with metabolic shift. In this study, we aim to pinpoint the metabolic pathway altered and assess its therapeutic potential.

**Methods:**

In silico reproduction of TCGA ovarian cancer dataset and functional annotation using GSEA was performed. Candidate metabolic pathway was validated using in vitro and in vivo assays.

**Results:**

From TCGA database, we found that polyamine metabolism was significantly enriched in Cyclin E1‐driven OvCa. Expressions of SMS, SRM, and ODC1 were positively correlated with that of CCNE1, respectively. ODC1 and SMS expressions were significantly correlated with decreased immune infiltrates. PGC‐1α silencing significantly decreased invasion and migration in both OvCa cell lines. Both spermidine and spermine levels were significantly increased when PGC‐1α was silenced. Targeting SRM significantly decreased spermine level in OVCAR3 cells, which was rescued when PGC‐1α was silenced. Silencing of PGC‐1α resulted in increased SRM in both OvCa cells. Dinaciclib significantly decreased invasion and migration of OVCAR3 cells. Expressions of PD‐L1 and PD‐L2 were predominantly in tumor‐infiltrating lymphocytes. Dinaciclib showed no notable effect of PD‐1 yet substantially induced the increased levels of PD‐L1 and PD‐L2.

**Conclusion:**

Cyclin E1‐driven OvCa is characterized with activated polyamine synthesis, which is associated with decreased cancer immunity. Targeting polyamine and CDK2 may therefore sensitize this genotype to immune checkpoint blockade.

## Graphical abstract

Cyclin E1‐driven ovarian cancer shows metabolic shift that is mediated by GCN5/PGC‐1α axis. Combination therapy targeting both CDK2 and GCN5 hold promise for combating Cyclin E1‐driven ovarian cancer.

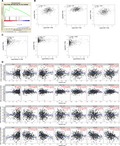

## 1 INTRODUCTION

Epithelial ovarian cancer (OvCa), a histopathologically, morphologically, and molecularly heterogeneous group of neoplasms.^1^ It is the fifth leading cause of cancer deaths among women in the developed world and the most lethal gynecological malignancy.^2^ Greater than 75% of ovarian cancer cases go undetected until an advanced stage, which is difficult to treat effectively.^3^ Due to the poor prognosis, the identification of potential factors with therapeutic potential in OvCa may have important clinical implications.

Epithelial ovarian cancer is highly heterogeneous with different genetic subtypes conferring distinct prognosis. Besides well‐established truncal TP53 mutations, HGSOC is also characterized with the high frequency of CCNE1 amplification and resulting upregulation (~30%). This genotype is termed Cyclin E1‐driven HGSOC.^4^ Of OvCa with intact homologous recombination function, amplification of CCNE1, which encodes the cell‐cycle regulator cyclin E1, is the best‐characterized driver. CCNE1‐amplification or gain occurs in 20% of all high‐grade OvCa tumors and is associated with primary treatment resistance and reduced overall survival in the entity.^5^, ^6^ A recent study, however, shows that 19q12 amplified and nonamplified subsets of high‐grade serous ovarian cancer with the overexpression of cyclin E1 differ in their molecular drivers and clinical outcomes, indicating that Cyclin E1‐driven OvCa should be subgrouped by CCNE1 amplification or overexpression.^7^

Cancer immunity plays critical role in combating the disease. Contemporary understanding of the immune checkpoint blockade (ICB) has made a handful of refractory cancers controllable or even curable. Nevertheless, here are currently no approved immune therapies for ovarian cancer.^8^ Lack of satisfactory response rate and durable effect are bottlenecks to extend ICB to OvCa. Therefore, elucidating genetic modulation on cancer immunity, especially for driver genes, may have important implications.

In the current study, we focus on the noncanonical gene enrichment and functional annotation in CCNE1‐amplified OvCa. With in vitro and in vivo modeling of the genotype, we aim at breaking bottleneck of treatment dilemma of OvCa and our findings may hold promise to precision targeted therapy for OvCa.

## 2 MATERIALS AND METHODS

### 2.1 In silico analysis

The TCGA database of high‐grade serous ovarian cancer was utilized and analyzed using the cBioPortal platform.^9^

### 2.2 GSEA

The GESA tool was used to analyze gene enrichment in CCNE1‐amplified OvCa cases. GSEA‐3.0.jar software was used and gene sets (“c2.cp.kegg.v6.2.symbols.gmt” and “c7.all.v6.2.symbols.gmt[immunologic signatures]”) from the website of Broad Institute and ran it under the support of Java 8.0.^10^ We considered cases with CCNE1 amplified (z‐score threshold: ±2) as group “CCNE1 altered” and the rest as “CCNE1 unchanged”.

### 2.3 TIMER

Correlations between gene expressions and immune infiltrates were studied using the TIMER (Tumor Immune Estimation Resource, https://cistrome.shinyapps.io/timer/) platform.^11^, ^12^

### 2.4 GEPIA

Correlations between the expressions of genes and “Exhausted T cells” gene signature were analyzed using the GEPIA platform (http://gepia.cancer-pku.cn/).^13^ The signature included HAVCR2, TIGIT, LAG3, PDCD1, CXCL13, and LAYN.

### 2.5 Cell lines

Cyclin E1‐driven ovarian cancer cell lines (OVCAR‐3 and A2780) were selected from the COSMIC database (http://cancer.sanger.ac.uk/cancergenome/projects/cosmic/) and obtained from the cell bank of Chinese Academy of Science. Both cell lines were cultured in complete RPMI‐1640 media supplemented with fetal bovine serum. Transcripts for shRNA construction targeting CCNE1, SRMand PGC‐1α (PPARGC1A) are selected from the The RNAi Consortium (TRC, http://www.broadinstitute.org/rnai/public/). Two transcripts for each gene were selected for testing (CCNE1: TRCN0000045299 and TRCN0000045298; SRM: Clone IDs TRCN0000045728 and TRCN0000290713; PPARGC1A: Clone IDs TRCN0000364085 and TRCN0000001165). Vectors with resistance to puromycin were constructed for knockdown and wild‐type paired cell lines via nonlipofectamine Fugene transfection. Medium was replaced with complete medium supplemented with 1:5000 of puromycin and changed every 3 days until all clones were negative for CDK2 or PPARGC1A expression. Similar methods were used to generate control cells.

### 2.6 Polyamine detection and quantification

The liquid chromatographic approach was used and chromatographic plate was prepared as per protocol. Standard polyamine solution was prepared using spermine, spermidine, cadecine, and putrescine dissolved in 5% perchloric acid at 10, 1, 0.1, and 0.01 mmol/L, respectively. Cell pellets were processed chilled perchloric acid and were centrifuged for supernatant. The crude extracts underwent sulfonation and extraction followed by chromatography. The spots of the polyamine derivative were scraped off and dissolved in 5 mL of ethyl acetate. The supernatant was subjected to fluorescence spectrophotometry. Relative absorbance was normalized for comparison.

### 2.7 mRNA expression and western blotting

Total RNA was extracted with Trizol reagent and was converted to cDNA. Primers for CCNE1, PPARGC1A, and SRM were designed using the PrimerBank (https://pga.mgh.harvard.edu/primerbank/) and corresponding primers were listed as follows: CCNE1 (forward, AAGGAGCGGGACACCATGA; reverse, ACG GTC ACG TTT GCC TTC C), PPARGC1A (forward, TCT GAG TCT GTA TGG AGT GAC AT; reverse, CCA AGT CGT TCA CAT CTA GTT CA) and SRM (forward, GTGGTGGCCTATGCCTACTG; reverse, CTCCTGGAAGTTCGTGCTCG). The relative expression level of each gene was determined by the real‐time quantitative PCR using SYBR Premix Ex Taq II (Takara). For western blotting, a standard protocol was followed. Total protein was first extracted and concentration was then determined. Proteins were then separated by SDS‐PAGE on a 10% gel and subsequently transferred to a polyvinylidene difluoride membrane. Nonspecific antigens were blocked using nonfat milk and the corresponding primary antibodies were applied according to the manufacturer's recommended protocol and concentration as follows: anti‐PD‐1 (Abcam, 1:100), anti‐PD‐L1 (Sigma‐Aldrich, 1:100), anti‐PD‐L2 (R&D, 1:100), anti‐SRM (ThermoFisher, 1:500), anti‐SMS (ThermoFisher, 1:200), anti‐ODC1 (Sigma‐Aldrich, 1:1000), anti‐MYC (ThermoFisher, 1:200), anti‐PGC‐1α (Santa Cruz Biotechnology, 1:100), anti‐Cyclin E1 (ThermoFisher, 1:1000) and ß‐catenin (Santa Cruz Biotechnology, 1:200). After corresponding secondary antibodies were applied, protein bands were then detected using chemiluminescence and autoradiography for densitometry detection.

### 2.8 Transwell assays

Transwell assay was used to study cell migration and invasion. Briefly, cells were resuspended at the density of 1 × 10^6^/mL in 300 μL of serum‐free medium and seeded in the upper chamber of Transwell without any coating for migration and with Matrigel coating for invasion. The lower chambers were then filled with 500 μl of complete medium and cells migrated through the membrane were stained with crystal violet and counted.

### 2.9 Statistical analysis

Comparisons between groups were analyzed with the two‐tailed Student's *t* test. The *P* value of .05 was accepted as statistically significant.

## 3 RESULTS

### 3.1 Polyamine metabolism is upregulated in Cyclin E1‐driven OvCa

**Figure 1:**
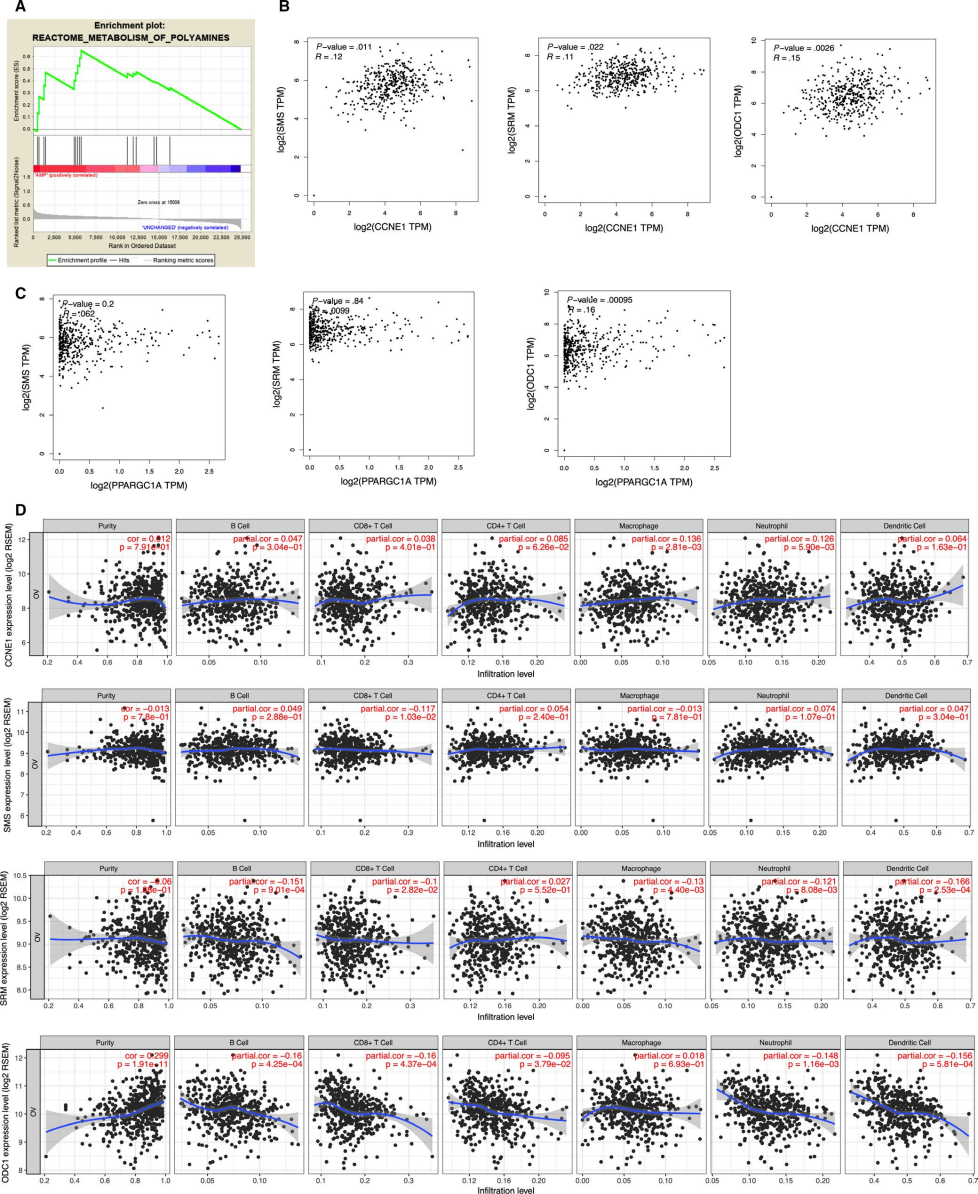
Polyamine metabolism is upregulated in Cyclin E1‐driven OvCa. Reproduced from TGCA OvCa database using (A) GSEA showing enrichment of polyamine metabolic genes; (B) GEPIA showing correlations between expressions of CCNE1 and polyamine genes; (C) GEPIA showing correlations between expressions of PGC‐1α and polyamine genes; and (D) TIMER showing correlations between expressions of genes

Using robust sequencing data of tissue samples from TCGA database, we found that polyamine metabolism was significantly enriched in Cyclin E1‐driven OvCa (Figure 1A). We then studied the correlation of expressions between CCNE1 and essential genes involved in polyamine metabolism and found that the expressions of SMS, SRM, and ODC1 were positively correlated with that of CCNE1, respectively (Figure 1B). We previously reported that PGC‐1α was downstream of Cyclin E1 and PGC‐1α was reported to regulate polyamine synthesis in prostate cancer.^14^, ^15^ We therefore queried the expressions of PPARGC1 against polyamine genes and found that the expressions of SMS, SRM, and ODC1 were positively correlated with that of CCNE1, respectively (Figure 1C). As polyamine was reported to mediate microenvironment and cancer immunity, we queried the expressions of CCNE1 and polyamine genes again immune infiltrates and found that the CCNE1 expression was significantly correlated with macrophage and neutrophil infiltration (Figure 1D). For polyamine genes, SMS expression was not correlated with immune infiltrates, whereas expressions of both ODC1 and SMS were significantly correlated with the decreased infiltration of B cell, CD8^+^ cells, macrophage, neutrophil and dendritic cells, respectively (Figure 1D). Here we show polyamine pathway was enriched in Cyclin E1‐driven OvCa we next tried to pinpoint the polyamine gene downstream of CCNE1.

### 3.2 PGC‐1α suppresses polyamine synthesis in Cyclin E1‐driven OvCa

**Figure 2:**
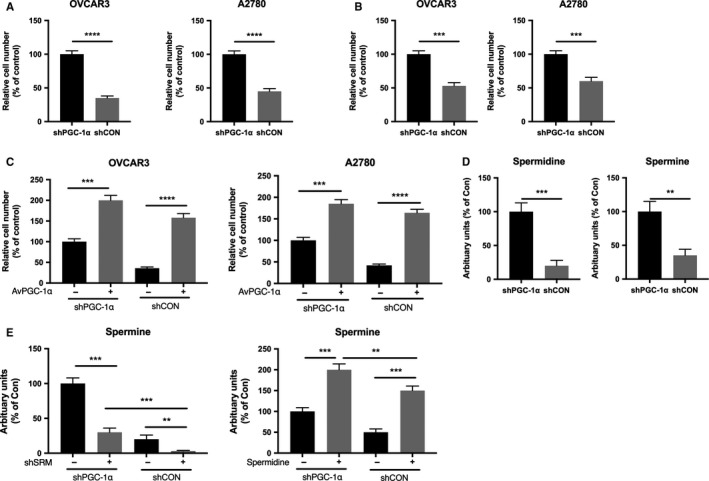
PGC‐1α suppresses polyamine synthesis in Cyclin E1‐driven OvCa. Shown were (A) invasion assay and (B) migration assay in OvCa cells with PGC‐1α silencing; (C) invasion assay demonstrating supplement of PGC‐1α using adenovirus on cells with or without PGC‐1α silencing; (D) levels of spermidine and spermine in response to PGC‐1α silencing in OVCAR3 cells; (E) Level of spermine in response to SRM silencing or spermidine supplement in OVCAR3 cells (**P < .01;***P < .001;****P < .0001)

To validate the effect of PGC‐1α on polyamine synthesis, we performed a series of Transwell assays to profile cancer invasiveness. We first found PGC‐1α silencing significantly decreased invasion and migration in both OvCa cell lines (Figure 2A,B). Supplement of PGC‐1α with adenovirus restored invasion in both cells (Figure 2C). Both spermidine and spermine levels were significantly increased when PGC‐1α was silenced in OVCAR3 cells (Figure 2D). Targeting SRM significantly decreased spermine level in OVCAR3 cells, which was rescued when PGC‐1α was silenced (Figure 2E). PGC‐1α silencing significantly increased spermine level which was further increased in the presence of spermidine (Figure 2E).

### 3.3 Targeting polyamine metabolism suppresses Cyclin E1‐driven OvCa

**Figure 3:**
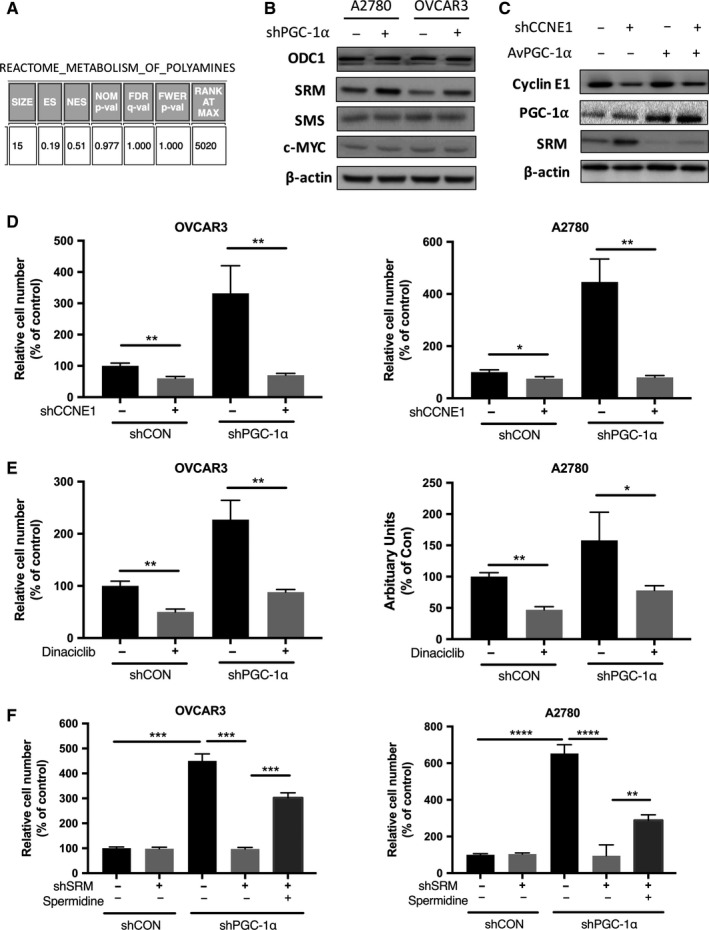
Targeting polyamine metabolism suppresses Cyclin E1‐driven OvCa. A, GSEA showing polyamine gene enrichment in MYC‐amplified cases in TCGA; B, effect of PGC‐1α silencing on polyamine genes in OvCa cell lines; C, effect of CCNE1 silencing and PGC‐1α overexpression on polyamine gene SRM in OVCAR3 cells; shown were invasion assays in OvCa cells with (D) CCNE1 and PGC‐1α silencing; E, Dinaciclib and PGC‐1α silencing; and (F) SRM, PGC‐1α silencing, and spermidine supplement (*P < .05; **P < .01; ***P < .001; ****P < .0001)

As MYC was reported to mediate polyamine metabolism^16^ and MYC was frequently amplified in OVCA and overlapped in part with CCNE1‐amplified cases, we first queried MYC‐amplified case in TCGA cohort and found that polyamine metabolism was not enriched (Figure 3A). Silencing of PGC‐1α resulted in increased SRM in both OvCa cells yet showed minimal effect on ODC1 or SMS (Figure 3B). Silencing of CCNE1 induced increased SRM level and silencing of PGC‐1α blocked the effect (Figure 3C). CCNE1 silencing significantly decreased the invasion and migration of OVCAR3 cells, whereas PGC‐1α silencing abolished the effect (Figure 3D). Similarly, CDK2 inhibitor Dinaciclib significantly decreased the invasion and migration of OVCAR3 cells, whereas PGC‐1α silencing abolished the effect (Figure 3E). Targeting SRM significantly inhibited the invasion of both OvCa cell lines which could be restored by spermidine supplement or PGC‐1α silencing (Figure 3F).

### 3.4 Targeting CDK2 induces PD‐L1 upregulation in tumor cells

**Figure 4:**
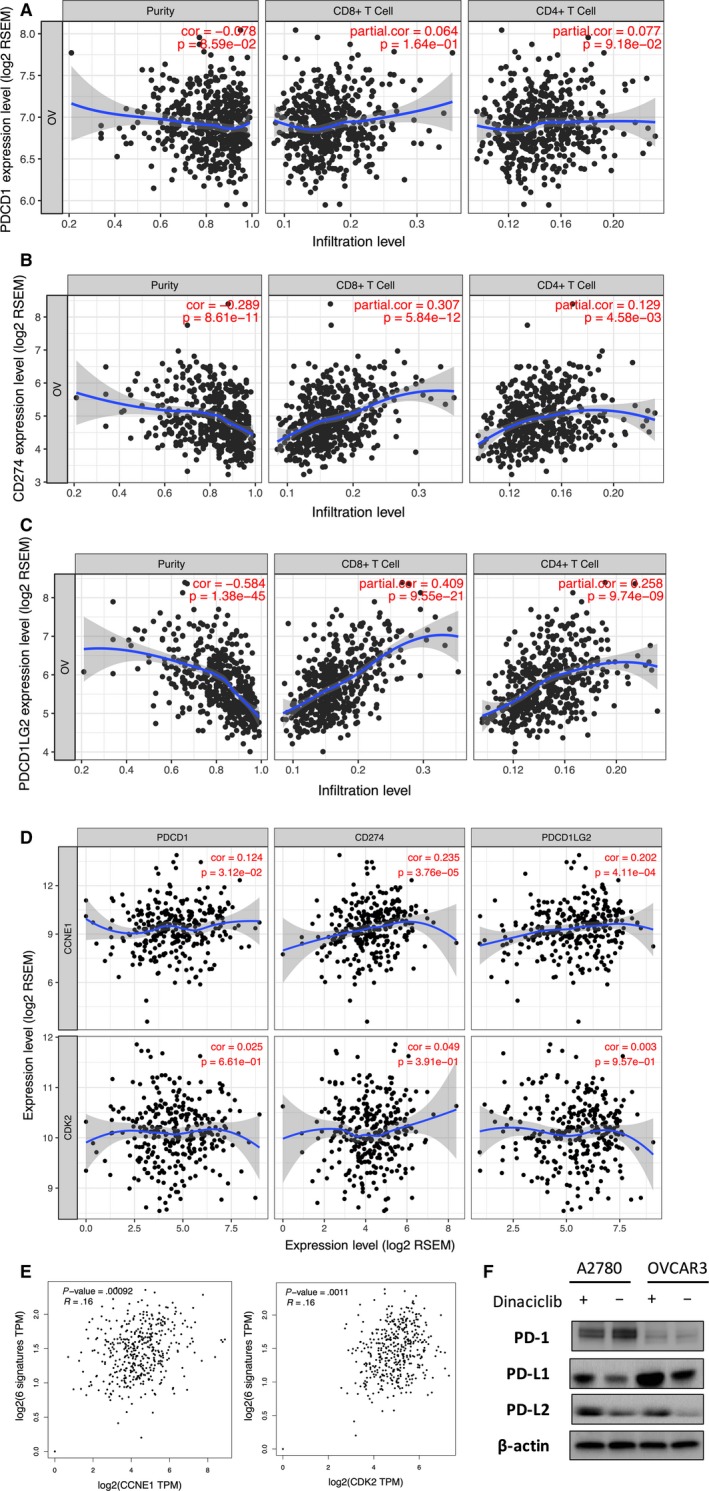
Targeting CDK2 induces PD‐L1 upregulation in tumor cells. Reproduced from TGCA OvCa database using TIMER showing correlations between tumor purity and expressions of (A) PD‐1; (B) PD‐L1; and (C) PD‐L2; and showing expressions between genes; Reproduction using GEPIA showing expressions of CCNE1 and CDK2 in association with exhausted T‐cell signature genes; (D) western blotting showing effect of Dinaciclib on immune checkpoint molecules in both OvCa cells; (E) correlations of expression of genes within "Exhausted T cell siguature" from GEPIA platform with expressions of CCNE1 and CDK2, respctively; (F) western blotting showing effect of Dinaciclib on levels of immune checkpoint molecules

As polyamine was reported to mediate cancer immunity, we here studied its impact on immune checkpoint molecules. Expression of PD‐1 was predominantly in tumor cells showing no correlation with CD4^+^ or CD8^+^ infiltrates (Figure 4A). Expressions of PD‐L1 and PD‐L2 were predominantly in tumor‐infiltrating lymphocytes and showed significant negative correlations with CD4^+^ or CD8^+^ infiltrates, respectively (Figure 4B,C). CCNE1 but not CDK2 expression showed significant positive correlation with the expressions of PD‐1, PD‐L1, and PD‐L2, respectively (Figure 4D). Expressions of CCNE1 and CDK2 were positively correlated with the gene signature of exhausted T cells (Figure 4E). Application of Dinaciclib in OvCa cells showed no notable effect of PD‐1 yet substantially induced the increased levels of PD‐L1 and PD‐L2 (Figure 4F).

## 4 DISCUSSION

In the current study, we have shown that polyamine metabolism is upregulated in Cyclin E1‐driven OvCa. PGC‐1α suppresses polyamine synthesis in Cyclin E1‐driven OvCa. Targeting polyamine metabolism suppresses Cyclin E1‐driven OvCa. Targeting CDK2 induces PD‐L1 upregulation in tumor cells. Modulation of polyamine metabolism by CCNE1 has not been reported before and has previously been mechanistically unrelated. Our findings further direct Cyclin E1 signaling to tumor microenvironment and notably, the immune checkpoint signaling. Cyclin D‐CDK4 kinase has been reported to destabilize PD‐L1 via cullin 3‐SPOP to control cancer immune surveillance.^17^ Together with our findings, it is highly possible that PD‐L1 protein stability can be regulated by a cell cycle kinase, revealing the potential for using combination treatment with CDK2 inhibitors and PD‐1‐PD‐L1 ICB to enhance therapeutic efficacy for OvCa.

Polyamine plays a role in cancer. The polyamines putrescine, spermidine, and spermine are polycationic alkylamines and are present in mammalian cells in millimolar concentrations.^18^ The direct interplay between oncogenes and polyamine metabolism was first apparent with the demonstration that ODC was a transcriptional target of the MYC oncogene.^19^ RAS activation has been associated with increased polyamine transport by colon tumor cells.^20^ It has also been demonstrated, in a series of human hepatocellular carcinomas and colon carcinoma cell models, that polyamine depletion through the overexpression of SSAT is a result of decreased AKT signaling and reduced nuclear β‐catenin leading to decreased cell growth, migration, and invasion.^21^ The PTEN–PI3K–mTOR complex 1 (mTORC1) pathway has been shown to be linked with polyamine metabolism in prostate cancer through the upregulation of AMD1.^22^ A noncanonical Hedgehog signaling pathway has recently been implicated in the upregulation of polyamine biosynthesis in the precursor lesion to medulloblastoma.^23^

Polyamine has also been associated with cancer immunity. Polyamine‐blocking therapy is reported to promote the antitumor immune response, resulting in even greater antitumour effects than would be expected from polyamine depletion in tumor cells alone. In immune‐competent mouse models of lymphoma, melanoma, and colon cancer, treatment with the combination of DFMO plus AMXT 1501 led to a decrease in tumor‐infiltrating myeloid suppressor cells and an increase in CD3^+^ T cells, resulting in inhibition of tumor growth.^24^ ODC activity and polyamines favor the tumor‐tolerant M2‐like phenotype while reducing the antitumor M1‐like phenotype. These findings are consistent with earlier work implicating ODC in the regulation of M1 macrophages.^25^

In the current study, we have also found that increased polyamine gene expression is associated with less immune infiltrates. We have further pursued correlation between polyamine and immune checkpoint molecules. Based on our findings, we speculate that Cyclin E1‐driven, in particular CCNE1‐amplified OvCa exert immune exclusion mainly via expression PD‐L1/L2 in TILs. Positive correlation between expressions of CCNE1 and PD‐L1/L2 revealed in TCGA dataset may result from response to TIL in the microenvironment. This corresponds to the in vitro observation that when CDK2 inhibitor is applied, PD‐L1/L2 in tumor is upregulated as a mechanism of resistance to would be decreased negative immune modulation. Although those speculations warrant validation, our findings hold promise for optimizing current ICB application in OvCa. Both polyamine deprivation and CDK2 inhibition is theoretically expected to sensitize cells to ICB.

## CONFLICT OF INTEREST

None.
